# A Her2-let-7-β2-AR circuit affects prognosis in patients with Her2-positive breast cancer

**DOI:** 10.1186/s12885-015-1869-6

**Published:** 2015-11-02

**Authors:** Dan Liu, Que Deng, Limin Sun, Tao Wang, Zhengyan Yang, Hongyu Chen, Liang Guo, Yanjun Liu, Yuanfang Ma, Ning Guo, Ming Shi

**Affiliations:** 1 Institute of Basic Medical Sciences, Beijing, 100850 P.R. China; 2 307 Hospital of People’s Liberation Army, Beijing, 100071 P.R. China; 3 Laboratory of Cellular and Molecular Immunology, Medical School of Henan University, Kaifeng, 475004 P.R. China

**Keywords:** β2-AR, Her2, let-7f, Breast cancer, Prognosis

## Abstract

**Background:**

Our previous studies show that β2-adrenergic receptor (β2-AR) is highly expressed in most Her2-overexpressing breast cancers. However, the mechanisms underlying upregulation of the β2-AR expression in Her2-overexpressing breast cancer cells are not fully understood. The clinical significance of the β2-AR overexpression in breast cancer is unclear.

**Methods:**

Human breast cancer cells MCF-7 and MCF-7/Her2 were transfected with the let-7 mimics or inhibitors. The expression of β2-AR was analyzed by Western blot. The β2-AR status in primary and metastatic sites of breast cancer and the human breast cancer tissue microarrays containing 49 primary tumors and 50 metastatic lymph node tissues was analyzed by immunohistochemistry. The correlation of lymph node metastasis with the β2-AR level was determined in 59 primary tumor tissues from the patients with Her2-positive breast cancer. The clinical prognostic significance of the β2-AR overexpression in the patients with Her2-positive breast cancers was evaluated by a retrospective study.

**Results:**

The let-7f level in Her2-overexpressing breast cancer cells SKBR3 and BT474 was significantly lower than that in MCF-7 cells, which express low level of Her2. Ectopic expression of Her2 in MCF-7 cells (MCF-7/Her2) represses the expression of microRNA let-7f, which is previously identified to regulate baseline β2-AR expression. The treatment with MEK1/2 inhibitors PD98059 or PD184352 effectively restored the let-7f level, suggesting that Her2-overexpression-mediated ERK constitutive activation inhibited let-7f, leading to the upregulation of the β2-AR expression. The transfection with the let-7f mimics markedly downregulated the β2-AR level, whereas the let-7 inhibitor significantly upregulated the β2-AR expression in both parental MCF-7 and MCF-7/Her2 cells. In addition, treatment of MCF-7/Her2 cells with isoproterenol resulted in a concentration-dependent reduction of the let-7f expression, demonstrating that the inhibitory effect of Her2 overexpression on let-7f can be reinforced by agonist-triggered β2-AR activation. We further demonstrate that high level of β2-AR associates with lymph node metastasis and poor outcome in the patients with Her2-positive breast cancer.

**Conclusions:**

The mutual and reciprocal interaction between Her2, β2-AR, and let-7f may maintain a high level of β2-AR in breast cancer cells. Our data suggest that β2-AR may be a new useful biomarker for predicting prognosis in Her2-positive breast cancer and may also be a promising selective therapeutic target for the aggressive subtype of breast cancer.

**Electronic supplementary material:**

The online version of this article (doi:10.1186/s12885-015-1869-6) contains supplementary material, which is available to authorized users.

## Background

Breast cancer is the most common malignancy and the second leading cause of cancer death in women. During recent decades, the incidence of breast cancer among women has been increasing throughout the world. In approximately 25 % of breast cancers Her2 is overexpressed. Overexpression of Her2 protein and/or amplification of Her2 gene play important roles in the development and progression of aggressive breast cancer and are correlated with unfavorable prognosis.

Her2 is a transmembrane tyrosine kinase receptor and belongs to the epidermal growth factor receptor (EGFR) family. It functions as a common co-receptor for other members of the EGFR family. Activation of Her2 through homodimerization or heterodimerization upon ligand binding triggers a cascade of its downstream events, eventually leading to activation of multiple signaling pathways including Ras/Raf/mitogen-activated protein kinase (MAPK) and phosphatidylinositol-3-kinase (PI3K)/Akt pathways, which critically regulate rapid growth, survival, and migration of tumor cells and confer resistance to the anticancer agents in breast cancer [1, 2].

Her family protein-mediated signaling can integrate heterologous signaling network. Our previous studies reveal that crosstalk of Her2 and β2-adrenergic receptor (β2-AR), an important member of seven transmembrane G protein-coupled receptors (GPCRs) [3, 4], triggers a stronger or more sustained biological effect in response to catecholamine stimulation. Activation of β2-AR by catecholamine promotes the expression of numerous pro-survival, invasion, angiogenesis, and metastasis genes, such as matrix metalloproteinases (MMPs), vascular endothelial growth factor (VEGF), hypoxia inducible factor-1α, MUC4, and CD44, through transactivating the extracellular signal regulated kinase (ERK), PI3K/Akt, and the mammalian target of rapamycin (mTOR) signaling [5–7].

Several studies including ours showed that β2-AR is overly expressed in a variety of tumor tissues, including ovarian, breast, prostate, and gastric cancers and catecholamines manipulate the biobehaviors of tumor cells mainly through activation of the β2-AR-mediated signaling pathways [3, 4, 8–11]. In our previous study, we demonstrated that chronic catecholamine stimulation induces the Her2 expression via activating STAT3 and promoting its binding to the *Her2* promoter. We also showed that excessive phosphorylation of ERK in Her2-overexpressing breast cancer cells upregulates the level of β2-AR. The interplay between β2-AR and Her2 may result in an enhanced mitogenic effect [3].

A recent study indicated that *ADRB2*, the gene encoding β2-AR is a target of microRNA (miRNA) let-7f. A conserved 8-nucleotide seed region was identified at the *ADRB2* 3′ UTR, with which the let-7 family can functionally interact. Targeting the specific region of the *ADRB2* 3′ UTR by the let-7 family leads to translational repression of β2-AR [12]. The let-7 family is known as a key regulator of cell proliferation and differentiation and a tumor suppressor by regulating multiple oncogenic signaling pathways. Deregulated expression of the let-7 family members has been linked to increased tumorigenicity and poor patient prognosis in several cancers, including breast cancer [13].

It has been suggested that the MAPK/ERK pathway modulates the miRNA-generating complex. Inhibition of the MAPK/ERK pathway enhanced the expression of let-7 [14]. Our previous study demonstrated that enforced overexpression of Her2 in breast cancer cells upregulated the expression of β2-AR at both mRNA and protein levels [3], raised a question as to how the expression of β2-AR is modulated by Her2 in breast cancer. We hypothesized that constitutive activation of ERK downregulates the expression of let-7f in the Her2-overexpressing breast cancer cells, resulting in upregulation of the β2-AR level. In the present study, we investigated that the effect of Her2 on the expression let-7f and β2-AR in breast cancer cells and evaluated clinical significance of the β2-AR expression in prognosis prediction of the patients with Her2-overexpressing breast cancer. We revealed a novel mechanism of the β2-AR upregulation in Her2-overexpressing breast cancer and demonstrated that high level of β2-AR is associated with lymph node metastasis and poor prognosis in Her2-positive breast cancer patients.

## Methods

### Cell culture and treatment

Human breast cancer cell lines MCF-7, SKBR3, and BT474 are obtained from the American Type Culture Collection. The MCF-7/Her2 cells stably overexpressing Her2 were established in our laboratory as described previously [15]. MCF-7, MCF-7/Her2, and SKBR3 cells were cultured in RPMI 1640 containing 10 % fetal bovine serum (FBS). BT474 cells were cultured in DMEM containing 10 % FBS. The cells were cultured in humidified atmosphere containing 5 % CO_2_ at 37 °C. For the treatment with the β2-AR agonist, the cells were incubated overnight in a serum-free medium and then treated with 2.5 μM isoproterenol (ISO) (Sigma) for the indicated time points. To investigate the role of the ERK and PI3K signaling pathways in the regulation of the let-7 expression, MCF-7/Her2 cells were pre-treated with 25 μM PD98059 for 24 h, 1 μM PD184352 for 2 h, 0.5 μM GDC0941 for 2 h or DMSO (as a solvent control) and then the expression of let-7f was analyzed by real-time RT-PCR.

### Transient transfection

The inhibitors and mimics of let-7f were provided by GenePharma Co., Ltd. The sequence of let-7f mimics is UGAGGUAGUAGAUUGUAUAGUU and the sequence of let-7f inhibitors is AACUAUACAAUCUACUACCUCA. MCF-7 and MCF-7/Her2 cells were planted in 24-well plates and transfected with 9 and 27 pmol synthetic inhibitors or mimics of let-7f, respectively, using Lipofectamine™ RNAiMAX (Invitrogen) according to the manufacturer’s instructions.

### Western blot

The whole cell lysates were prepared, separated by SDS-PAGE, and transferred to PVDF membranes. After blocking, blots were probed with the appropriate primary antibodies overnight at 4 °C. The blots were then washed and incubated with horseradish peroxidase-conjugated secondary antibodies. Bands were detected by enhanced chemiluminesence (Pierce). The antibodies were used for immunoblotting: the antibodies against Her2 (4290, Cell Signaling), p-ERK (4370, Cell Signaling), ERK (4695, Cell Signaling), β2-AR (sc-569, Santa Cruiz), and glyceraldehyde-3-phosphate dehydrogenase (GAPDH, Sungene Biotech). All experiments were performed in duplicate.

### Real-time RT-PCR

The expression of let-7f was detected by real-time RT-PCR using Hairpin-itTM MicroRNAs Quantitation PCR kit (Genepharma) and MX3000p real-time PCR detection system following the manufacturer’s instruction (Genepharma). The experiments were performed three times independently.

### Immunohistochemistry

Immunohistochemical staining was performed as previously described [3]. To analyze the β2-AR status in primary and metastatic sites of breast cancer, the human breast cancer tissue microarray, containing 49 primary tumors and 50 metastatic lymph node tissues (one core per tumor/lymph node tissue), were purchased from the US Biomax company. The two consecutive sections of the microarray were used to evaluate the expression of Her2 or β2-AR. The mean scores of 2 cores from identical case in the two consecutive sections were taken.

Tissue sections were deparaffinized with xylene and rehydrated through a graded alcohol series and washed. To block the endogenous peroxidase activity, the sections was incubated with 3 % hydrogen peroxide for 10 min, followed by heat-induced antigen retrieval in 1 mM EDTA buffer pH 8.0 for 20 min. The sections were incubated with normal goat serum and then with the anti-Her2 (Cell Signaling Technology, 4290) or anti-β2-AR antibody (Abcam, ab13163) overnight at 4 °C. The slides were washed and then incubated with secondary antibody conjugated with horseradish peroxidase (ZSGB-BIO). The bound antibodies were visualized using diaminobenzidine chromogen (ZSGB-BIO). The slides were counterstained with haematoxylin. Western blot was employed to test the specificity of the primary antibodies. The staining was assessed microscopically by two independent pathologists. Images were taken on an Olympus BX51 microscope (Olympus) using the Spot insight image capture system CCD camera. An intensity proportion scale ranging from 0 to 3+ are used for scoring of β2-AR: 3+, greater than 30 % tumor cells strongly stained; 2+, greater than 30 % tumor cells modestly stained; 1+, greater than 15 % tumor cells weakly stained; 0, no staining or less than 10 % of tumor cells stained.

To investigate the correlation between the level of β2-AR and prognosis of the patients with breast cancer, the primary invasive breast cancer tissues from 29 patients with Her2 overexpression and prognosis-related information were obtained from 307 Hospital of People’s Liberation Army. The rates of disease-free survival (DFS) and overall survival (OS) were determined using the Kaplan-Meier analysis. To determine the correlation of lymph node metastasis (LNM) with the β2-AR level, immunohistochemical staining for β2-AR was performed on 59 primary tumor tissues from the patients with Her2-positive breast cancer. All tumor tissue samples and related information were obtained from 307 Hospital of People’s Liberation Army. The Her2 status in all tumor tissues was confirmed by either fluorescence in situ hybridization or immunohistochemistry.

Written informed consents were obtained from the patients for the use of the tumor tissue samples in this research. The study was approved by the ethics and scientific committee of 307 Hospital of People’s Liberation Army.

### Statistical analysis

For *in vitro* assays, the data were analyzed by ANOVA test and Student’s unpaired *t*-test. The survival was estimated by the Kaplan-Meier method and survival characteristics were compared using log rank tests. DFS was determined as an interval between the first day of therapy and the date of the development of progressive diseases. OS was measured from the date of therapy to the date of death or last follow-up. *P* < 0.05 was considered statistically significant. The distribution of LNM and no LNM cases in two groups was analyzed by Chi-square test.

## Results

### β2-AR is highly expressed in Her2-positive breast cancer

**Fig. 1:**
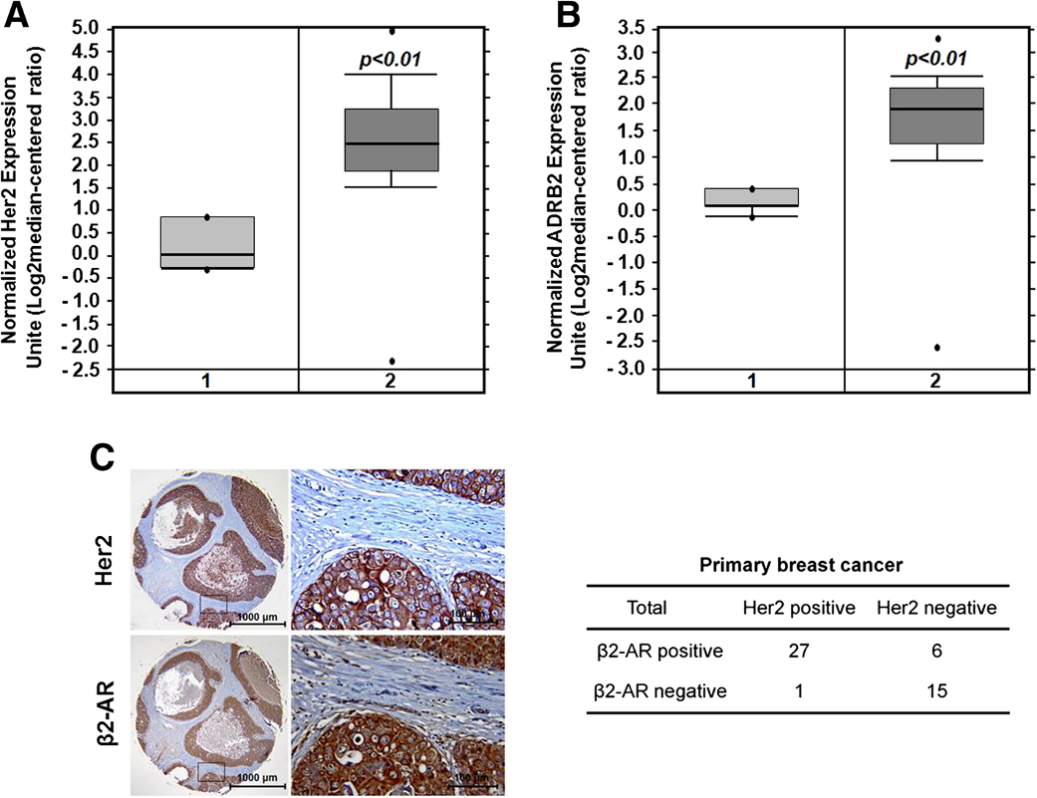
β2-AR is highly expressed in Her2-positive breast cancer tissues. **a** and **b**, The relative mRNA expression of Her2 (**a**) and *ADRB2* (**b**) in human breast cancer (2, *n* = 53) and normal breast tissue samples (1, *n* = 6) was analyzed by searching a publicly available database Oncomine (www.oncomine.org). **c**, The expression of Her2 and β2-AR was detected by immunohistochemistry on a human breast cancer tissue microarray consisting of 49 tumor tissues from breast cancer patients. Bar = 1000 μm (low-power field) or 100 μm (high-power field)

In our previous study, we demonstrate that enforced overexpression of Her2 in breast cancer cells upregulates the expression of β2-AR at both mRNA and protein levels [3], raised questions as to how the expression of β2-AR is modulated by Her2 in breast cancer. We interrogated the relative mRNA expression of *ADRB2* (β2-AR) in human breast cancer tissue samples by searching a publicly available database Oncomine (www.oncomine.org). In the majority (52/53) of the breast cancer tissue samples collected, Her2 is overexpressed. Coincidently, the levels of β2-AR mRNA are also high in these tumor tissues (Fig. 1a and b). Coexpression of Her2 and β2-AR at protein levels was further confirmed by immunohistochemistry on a human breast cancer tissue microarray consisting of 49 tumor tissues from breast cancer patients. Among the Her2-overexpressing tumor tissues, ~96 % (27/28) was β2-AR-positive (Fig. 1c). However, in Her2-negative tumors only ~29 % (6/21) was β2-AR-positive. 15 tumor tissues were double negative. The results were consistent with the findings in our previous study [3].

### Let-7f regulates β2-AR expression in breast cancer cells

**Fig. 2:**
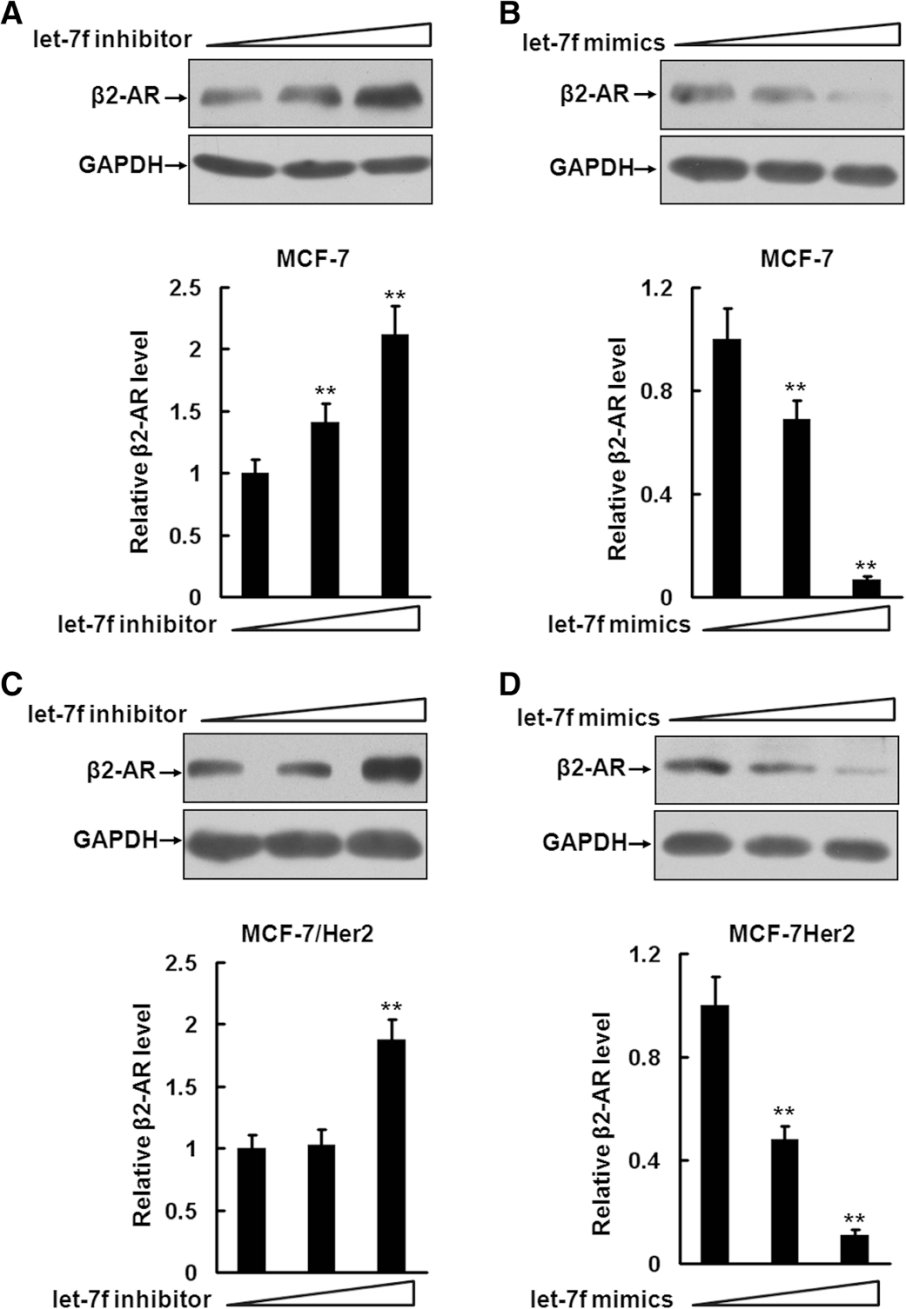
Let-7f regulates β2-AR expression in breast cancer cells. **a** to **d**, MCF-7 (**a** and **b**) and MCF-7/Her2 cells (**c** and **d**) were planted in 24-well plates and transfected with 9 and 27 pmol synthetic inhibitors or mimics of let-7f. The expression of β2-AR was analyzed by Western blot. These experiments were repeated twice

A recent study indicated that miRNA let-7f regulates baseline β2-AR expression [12]. In human airway epithelial cells, let-7f inhibits the β2-AR expression through a direct interaction with the 3′ UTR of the gene encoding β2-AR (*ADRB2*) that harbors a conserved 8-nucleotide seed region of let-7 family [12]. To determine whether let-7f regulates the expression of β2-AR and how the expression of β2-AR is upregulated in Her2-overexpressing breast cancer cells, we first established MCF-7/Her2 cells [15], which stably overexpress Her2 (Additional file 1: Figure S1). Then parental MCF-7 and MCF-7/Her2 cells were transfected with 9 and 27 pmol of synthetic mimics or inhibitors of let-7f. Figure 2a and c show that the treatment with the let-7 inhibitors caused a concentration-dependent increase of the β2-AR expression in both MCF-7 and MCF-7/Her2 cells. In contrast, the transfection with the let-7f mimics exhibited a marked inhibitory effect on the β2-AR expression in a concentration-dependent manner (Fig. 2b and d).

### Her2 overexpression inhibits let-7f via constitutive activation of ERK

Several recent studies indicated that the expression of the let-7 family is significantly downregulated in human cancers, including breast cancer. It has been reported that the copy number of let-7 family genes is reduced in breast cancer [13, 16]. Comparison of miRNA expression profiles using clinical breast cancer biopsies revealed that the expression of let-7f is significantly lower in Her2-positive than Her2-negative breast cancer [17].

**Fig. 3:**
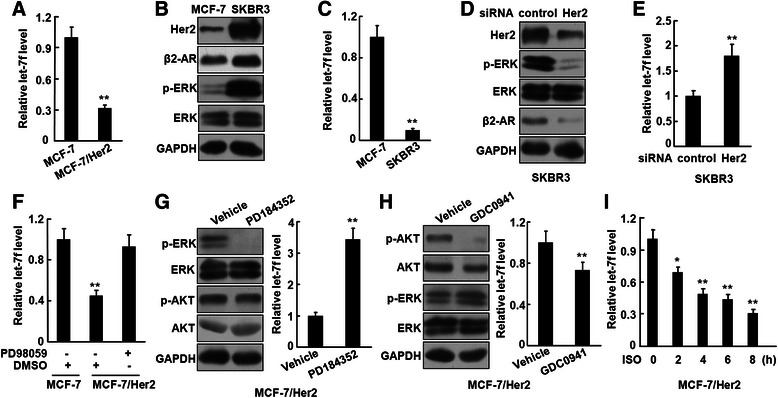
Her2 overexpression inhibits let-7f via constitutive activation of ERK. **a**, The expression of let-7f in MCF-7 and MCF-7/Her2 cells was detected by real-time RT-PCR. **b** and **c**, The expression of Her2, β2-AR, and phosphorylated ERK in MCF-7 and SKBR3 cells was analyzed by Western blot (**b**) and the level of let-7f was detected by real-time RT-PCR (**c**). **d** and **e**, SKBR3 cells were transfected with the siRNA targeting Her2. The expression of Her2, β2-AR, and phosphorylated ERK was analyzed by Western blot (**d**) and the level of let-7f was detected by real-time RT-PCR (**e**). **f**, MCF-7/Her2 cells were pre-treated with 25 μM PD98059 or DMSO (as a solvent control) for 24 h and the expression of let-7f was analyzed. **g** and **h**, MCF-7/Her2 cells were pre-treated with 1 μM PD184352 (**g**) or 0.5 μM GDC0941 (h) for 2 h. The levels of phosphorylated ERK and AKT were analyzed by Western blot and the expression of let-7f was detected by real-time RT-PCR. **i**, MCF-7/Her2 cells were treated with 2.5 μM ISO and the expression of let-7f was analyzed by real-time RT-PCR. These experiments were repeated at least twice. *P < 0.05; **P < 0.01

The expression of let-7 can be inhibited by mitogenic signaling-mediated ERK activation [14]. It is known that overexpression and homodimerization of Her2 result in the autophosphorylation of tyrosine residues within the cytoplasmic domain of the receptor and activation of ERK signaling pathway. As shown in Additional file 1: Figure S1, the overexpression of Her2 was companied by constitutive activation of ERK in MCF-7/Her2 cells. We assumed that the Her2 overexpression may affect the let-7 level in breast cancer cells. We examined the let-7f expression in MCF-7/Her2 cells by real-time RT-PCR. Fig. 3a demonstrates that the let-7f level was remarkably reduced approximately 3 folds, compared with the parental cells. In human breast cancer cell line SKBR3, which expresses high level of endogenous Her2, ERK was constitutively activated. Coincidently, the level of let-7f in SKBR3 cells is significantly lower than that in MCF-7 cells, in which both Her2 and phosphorylated ERK were at low levels (Fig. 3b and c). In addition, the expression of let-7a was also downregulated in MCF-7/Her2 and SKBR3 cells (Additional file 2: Figure S2A and S2B). Knockdown of the Her2 expression in SKBR3 cells not only importantly inhibited the β2-AR expression and ERK phosphorylation, but also increased the let-7f level (Fig. 3d and e). Similar data were obtained in BT474 cells overexpressing Her2 (Additional file 3: Figure S3A and S3B). The ERK and PI3K/Akt are two major Her2-mediated downstream signaling pathways. Inhibition of the ERK pathway by MEK1/2 inhibitors PD98059 or PD184352 effectively restored the let-7f level (Fig. 3f and g), but PI3K inhibitor GDC0941 did not, suggesting that Her2-mediated ERK activation inhibited the expression of let-7f. Interestingly, the let-7f level was somehow down-regulated after treatment with GDC0941 (Fig. 3h). We noticed that GDC0941 treatment slightly upregulated the level of phosphorylated ERK in MCF-7/Her2 cells. However, the mechanism underlying upregulation of ERK by GDC0941 is currently unexplained.

Our previous studies showed that Her2 transcription is upregulated by β2-AR-mediated Stat3 activation and that Her2 and its downstream signaling can be transactivated by β2-AR in response to catecholamine stimulation [3, 18], implicating that interplay between β2-AR and Her2 may influence the expression of let-7f. We investigated whether the β2-AR signaling interferes with the expression of let-7f by treating MCF-7/Her2 cells with 2.5 μM ISO. The treatment resulted in a time-dependent reduction of the let-7f expression (Fig. 3i). The data indicate that Her2 overexpression-induced ERK activation enhances the β2-AR expression by downregulating the level of let-7f and that the inhibitory effect of Her2 can be reinforced by agonist-triggered β2-AR activation. The mutual and reciprocal interaction between Her2, β2-AR, and let-7f may maintain a high level of β2-AR and a low level of let-7f in breast cancer cells.

### β2-AR overexpression correlates with DFS in breast cancer patients

**Fig. 4:**
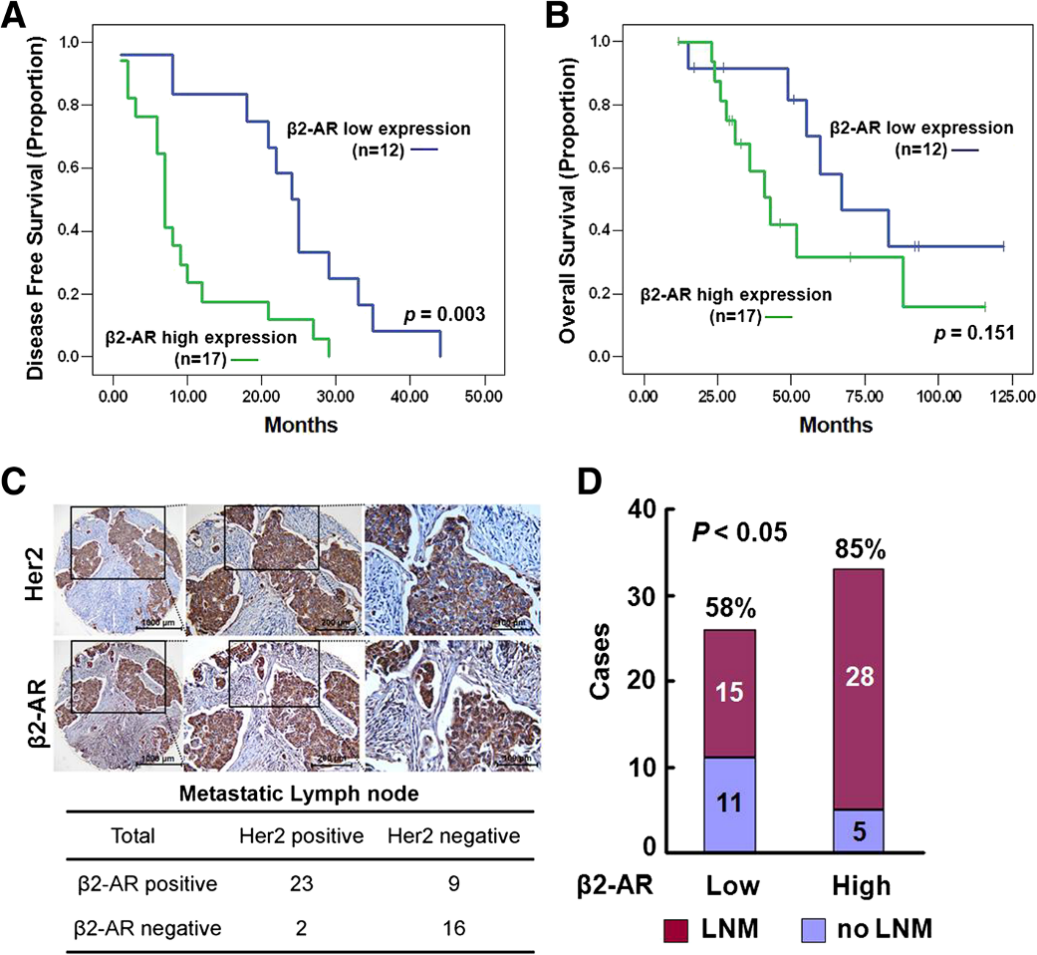
β2-AR overexpression correlates with DFS and LNM in breast cancer patients. **a** and **b**, The rates of DFS (**a**) and OS (**b**) in the patients with Her2-positive metastatic breast cancer according to the expression level of β2-AR were determined by the Kaplan-Meier analysis. **c**, The expression of Her2 and β2-AR was analyzed using a tissue microarray containing 50 metastatic lymph nodes from breast cancer patients by immunohistochemical staining. The middle and right panels are the magnifications of the square regions in the left and middle panels, respectively. Bar = 1000 μm (low-power field), 200 μm or 100 μm (high-power field). **d**, The relationship between LNM and β2-AR expression was evaluated in Her2-overexpressing breast cancer

Although Her2 overexpression represents a highly aggressive phenotype of breast cancer, the prognosis of the patients with Her2-overexpressing breast cancers may vary somehow. The biomarkers that can predict clinical outcome of the patients with Her2-overexpressing breast cancer are currently unknown [19]. It has been demonstrated that crosstalk between GPCRs and EGFR contributes to cancer malignant progression [20–22]. Therefore, we evaluated the clinical prognostic significance of the β2-AR overexpression in the patients with Her2-positive breast cancers by retrospectively investigating the relationship between the level of β2-AR and DFS or OS of the patients. The expression of β2-AR in the primary tumors from 29 Her2-positive breast cancer patients was assessed by immunohistochemistry. Immunostaining was scored as high (3+++) and low/moderate (0 – 2++) according to the rate of positive cells and staining intensity (Additional file 4: Figure S4). The rates of DFS and OS were determined using the Kaplan-Meier analysis. The level of β2-AR was high in 17 tumors. The patients with β2-AR-overexpressing tumors had a significantly lower DFS rate (*P* = 0.003, log-rank test; Fig. 4a). The OS rates at 5 years were 58.2 % for the low/moderate β2-AR group and 31.6 % for the high β2-AR group, but the difference between two groups was not statistically significant (*P* = 0.151, long-rank test; Fig. 4b). This could be due to the relatively short follow-up time and small sample size in this study. Nevertheless, these data implicate that the β2-AR overexpression correlates with poor prognosis in Her2-positive breast cancer.

### β2-AR overexpression correlates with LNM in breast cancer patients

The previous studies indicate that aberrant activation of the β2-AR-mediated signaling pathways promotes the malignant progression of cancer. Compelling evidence demonstrates that migrative, invasive, and metastatic capacities of cancer cells are critically regulated by the β2-AR-mediated signaling [6]. Thus, we examined the expression of β2-AR and Her2 in metastatic lymph nodes using a tissue microarray containing 50 metastatic lymph nodes from breast cancer patients. In agreement with the findings that Her2 and β2-AR were coexpressed in primary breast cancer tissues, the expression of β2-AR was also detected in most Her2-overexpressing metastatic lymph nodes (23/25, 92 %) as shown in Fig. 4c. We further evaluated the correlation of LNM with the expression of β2-AR in 59 Her2-overexpressing breast cancer patients (Additional file 5: Table S1). The incidence (28/33, 85 %) of LNM was significantly higher in the patients with high expression of β2-AR than in those patients with low/moderate expression of β2-AR (15/26, 58 %; *P* < 0.05; Fig. 4d). The data demonstrate that the β2-AR level significantly correlates with lymph node metastasis in Her2-positive breast cancer patients.

## Discussion

It is becoming increasingly clear that the β2-AR-mediated signaling plays a key role in the malignant progression of cancer [6, 11]. Catecholamines can stimulate the expression of multiple molecules involved in tumor cell proliferation, migration, invasion, adhesion, and metastasis, influencing biological behaviors of tumor cells [23]. It has been reported that the level of catecholamines is high in tumor microenvironment. Both tumor and nontumor cells may contribute to the increase of the catecholamine level in tumor microenvironment [6, 24, 25]. In tumor cells, β2-AR, which functions as an intermediary in transmembrane signaling pathways, mediates the effects of catecholamines.

Increasing evidence indicates that crosstalk between GPCR and growth factor receptors profoundly affect pathophysiological consequences of tumor progression. The findings in this study show that the β2-AR protein is overly expressed in most Her2-positive breast cancer tissues. The β2-AR mRNA level was also high in Her2-positive breast cancer. Our previous study shows that catecholamines promote β2-AR/Her2 complexation and induce β2-AR-mediated Her2 transactivation [18], implicating that reciprocal influence between Her2 and β2-AR may occur at transcriptional and posttranscriptional levels. Let-7f is a recently identified inhibitor of β2-AR. Analysis of miRNA expression profiling reveals that let-7f is significantly downregulated in Her2-positive breast cancer [17]. Our data demonstrate that constitutive ERK activation in the Her2-overexpressing breast cancer cells repressed the level of let-7f and that the inhibitory effect could be enhanced by the β2-AR agonist, indicating a novel mechanism of the β2-AR expression upregulation in Her2-overexpressing breast cancer. The interplay of the β2-AR- and Her2-mediated pathways synergistically abrogates the regulatory functions of the oncogene suppressor let-7 and maintains a high level of β2-AR in breast cancer.

Human breast cancer is a clinically heterogeneous disease, consisting of a variety of distinct subgroups of tumors with varying levels of gene and protein expression, which endow human breast cancer with different clinical characteristics, disease courses, and responses to specific treatments [26]. Based on genomic profiling, breast cancers are divided into several molecularly defined subtypes, including luminal A (ER/PR+, Her2-), luminal B (ER/PR/Her2+), Her2 (mostly Her2 amplified and ER-), normal-breast-like (the highest expression of the genes known to be expressed by adipose tissue and other nonepithelial cell types), and basal-like types (mostly ER-). These molecular subtypes allow for a more rational, patient-specific approach to therapy and prediction of clinical courses. We observed that high level of β2-AR was closely associated with LNM and poor DFS in Her2-positive breast cancer patients, indicating that β2-AR is a potential prognostic biomarker for survival and tumor recurrence in Her2-overexpressing breast cancers. A recent study showed that single nucleotide polymorphisms of the β2-AR gene were associated with LNM, poor prognosis, and high expression levels of β2-AR, EGFR, VEGF, and MMP-2 in pancreatic carcinoma [27]. The β2-AR expression was also associated with poor prognosis, tumor-node-metastasis stage, and Edmondson stage in hepatocellular carcinoma patients [28]. However, there is a contradictory report showing that strong β2-AR expression was an independent favorable prognostic factor for oral squamous cell carcinoma patients [29]. Further investigations are needed to determine whether β2-AR as a prognostic predictor is dependent upon certain types of cancers.

Combinations of different markers allow for the identification of tumors susceptible to targeted treatments. Generally, the subgroups with the Her2 expression have the shortest relapse-free and overall survival. However, Her2-positive breast cancers receive benefit from targeted therapies such as the monoclonal antibody trastuzumab, which binds to Her2 [30, 31]. Our recent study demonstrated that catecholamine-induced β2-AR activation mediates desensitization of gastric cancer cells to trastuzumab [4]. Several retrospective studies reported that β-blocker use reduced distant metastasis, tumor recurrence, and cancer specific mortality [32–35]. These data implicate that β2-AR may be used as a new therapeutic target to improve existing targeted therapies.

## Conclusions

β2-AR is predominantly expressed in most Her2-overexpressing breast cancers. Her2-mediated activation of ERK represses miRNA let-7f, leading to the upregulation of the β2-AR expression. High level of β2-AR associates with lymph node metastasis and poor outcome. β2-AR may be a new useful biomarker for predicting prognosis in Her2-positive breast cancer and may also be a promising selective therapeutic target for the aggressive subtype of breast cancer.

## Additional files

Additional file 1: Figure S1. The expression of Her2 and phosphorylation of ERK in parental MCF-7 and MCF-7/Her2 cells were analyzed by Western blot. (TIFF 819 kb)
Additional file 2: Figure S2. A and B, The expression of let-7a was analyzed in MCF-7, MCF-7/Her2 (A), and SKBR3 cells (B) by real-time RT-PCR. (JPEG 591 kb)
Additional file 3: Figure S3. A and B, BT474 cells were transfected with the siRNA targeting Her2. The expression of Her2, β2-AR, and phosphorylated ERK was analyzed by Western blot (A) and the level of let-7f was detected by real-time RT-PCR (B). (JPEG 803 kb)
Additional file 4: Figure S4. The expression of β2-AR in the primary tumors from Her2-positive breast cancer patients was assessed by immunohistochemistry with the antibody against β2-AR. H, high expression; M, moderate expression; L, low expression; Bar = 100 μm (JPEG 921 kb)
Additional file 5: Table S1. Patients and Tumor Characteristics. (DOC 47 kb)

## Abbreviations

β2-AR: β2-adrenergic receptor
EGFR: epidermal growth factor receptor
MAPK: mitogen-activated protein kinase
PI3K: phosphatidylinositol-3-kinase
GPCRs: G protein-coupled receptors
MMPs: matrix metalloproteinases
VEGF: vascular endothelial growth factor
ERK: the extracellular signal regulated kinase
mTOR: the mammalian target of rapamycin
miRNA: microRNA
FBS: fetal bovine serum
ISO: isoproterenol
DFS: disease-free survival
OS: overall survival
LNM: lymph node metastasis
