# Supplementary figures and images for: A Her2-let-7-β2-AR circuit affects prognosis in patients with Her2-positive breast cancer
Source: BMC Cancer. 2015 Nov 2;15:832. doi: 10.1186/s12885-015-1869-6 (PMC4629406; doi:10.1186/s12885-015-1869-6)

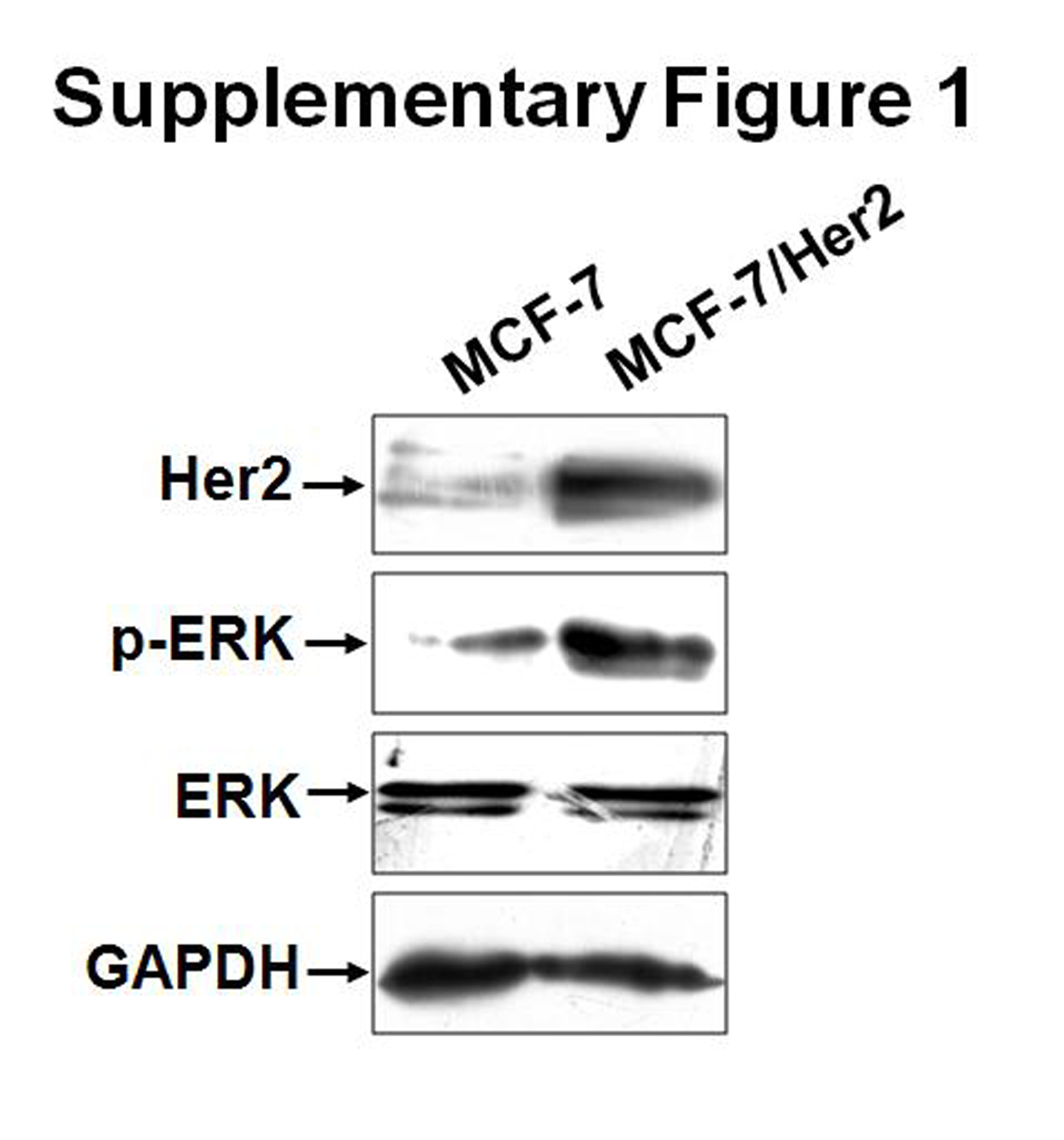

Supplement: Additional file 1: Figure S1. — The expression of Her2 and phosphorylation of ERK in parental MCF-7 and MCF-7/Her2 cells were analyzed by Western blot. (TIFF 819 kb) [file 12885_2015_1869_MOESM1_ESM.tiff]

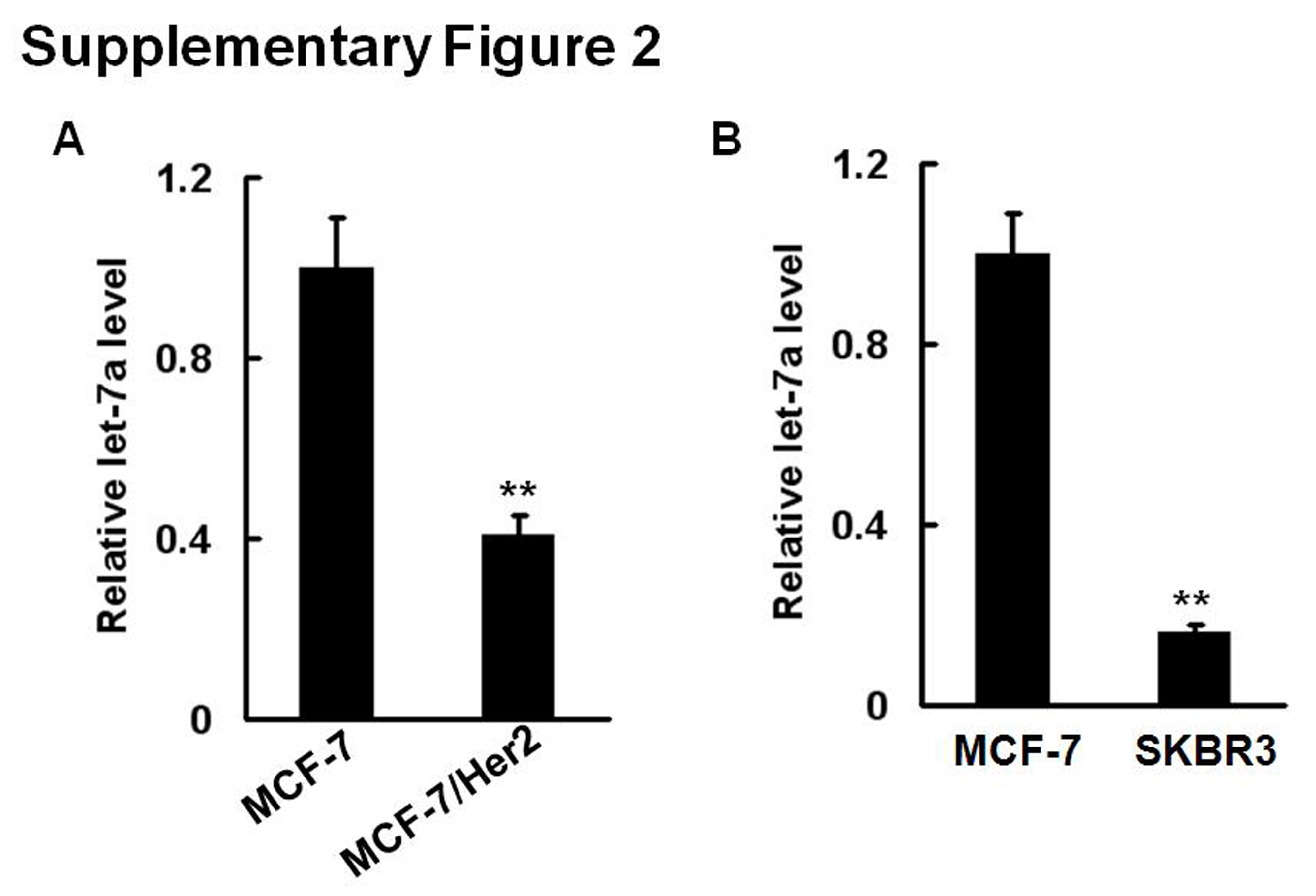

Supplement: Additional file 2: Figure S2. — A and B, The expression of let-7a was analyzed in MCF-7, MCF-7/Her2 (A), and SKBR3 cells (B) by real-time RT-PCR. (JPEG 591 kb) [file 12885_2015_1869_MOESM2_ESM.jpeg]

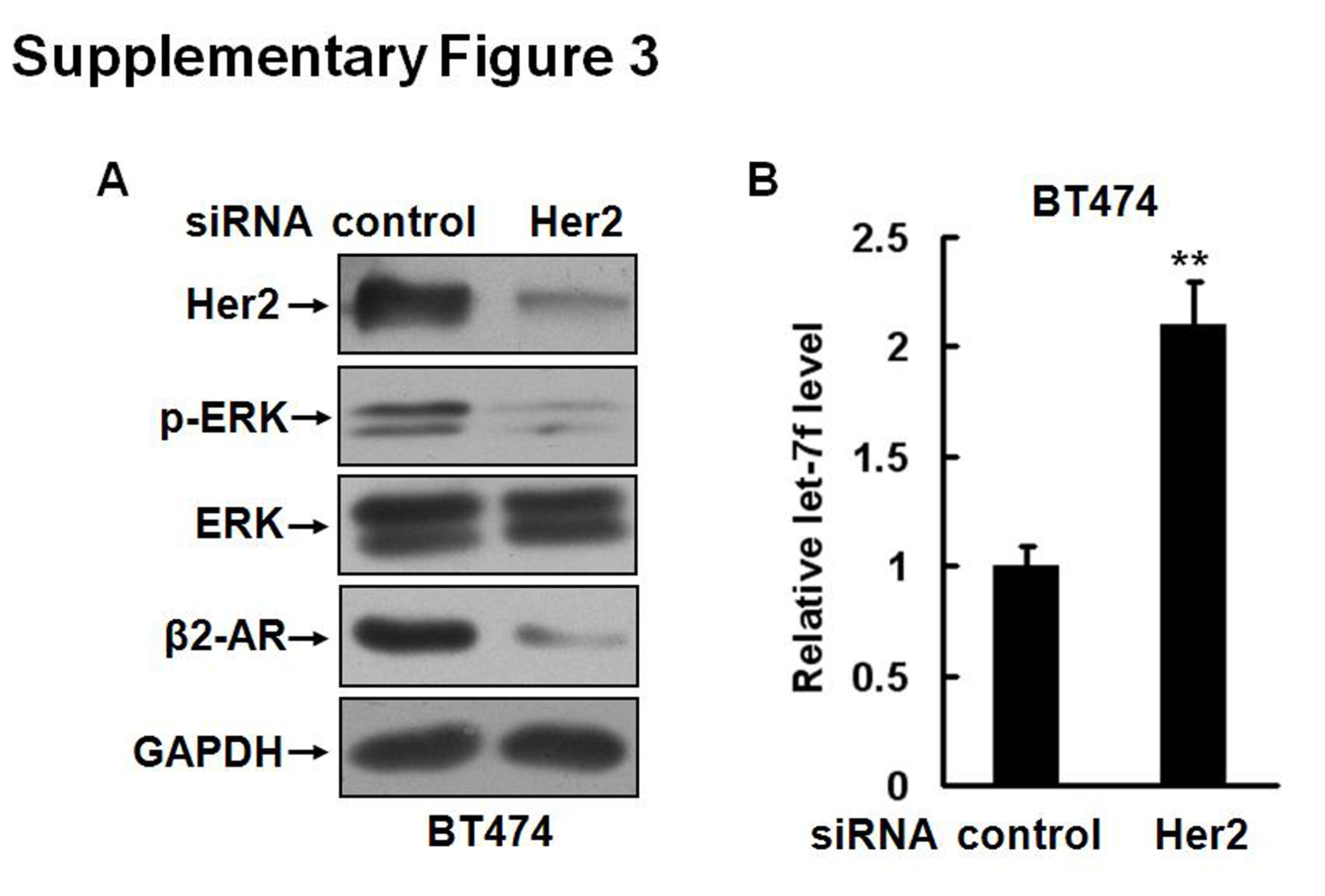

Supplement: Additional file 3: Figure S3. — A and B, BT474 cells were transfected with the siRNA targeting Her2. The expression of Her2, β2-AR, and phosphorylated ERK was analyzed by Western blot (A) and the level of let-7f was detected by real-time RT-PCR (B). (JPEG 803 kb) [file 12885_2015_1869_MOESM3_ESM.jpeg]

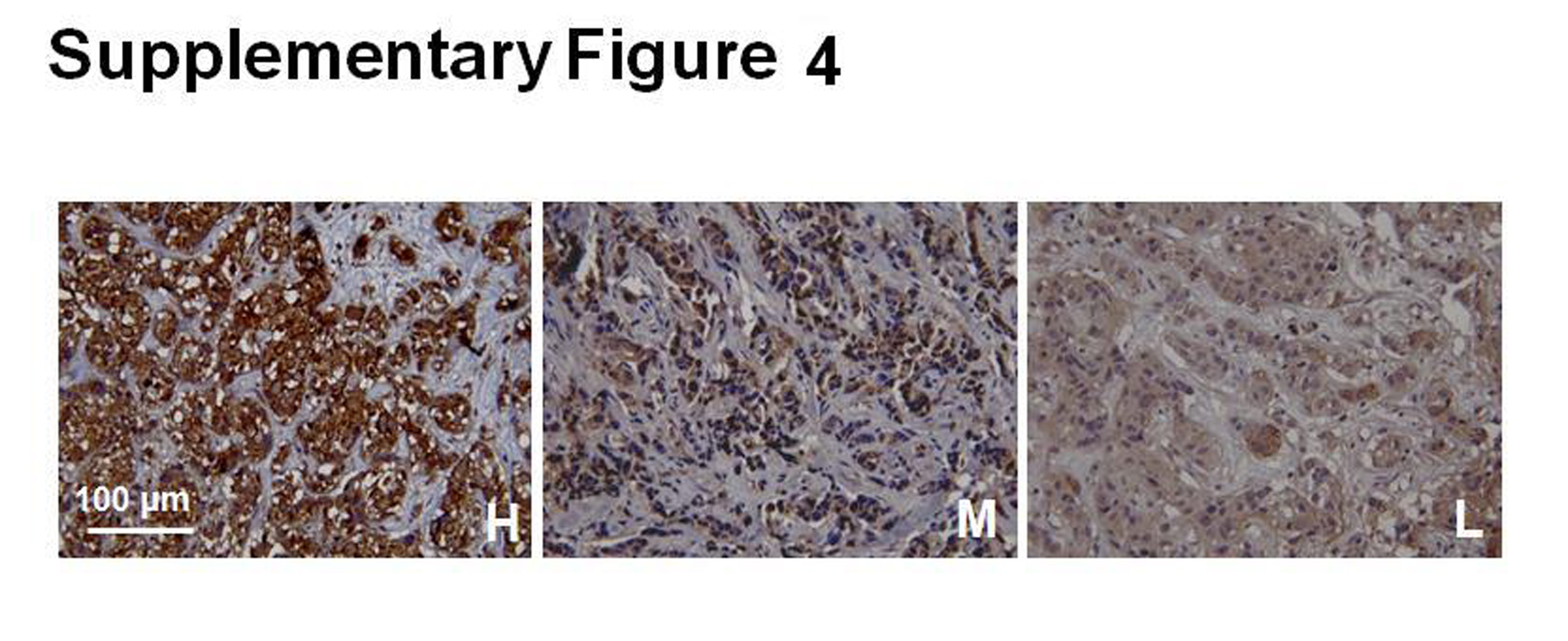

Supplement: Additional file 4: Figure S4. — The expression of β2-AR in the primary tumors from Her2-positive breast cancer patients was assessed by immunohistochemistry with the antibody against β2-AR. H, high expression; M, moderate expression; L, low expression; Bar = 100 μm (JPEG 921 kb) [file 12885_2015_1869_MOESM4_ESM.jpeg]
