# Supplementary material for: A Her2-let-7-β2-AR circuit affects prognosis in patients with Her2-positive breast cancer
Source: BMC Cancer. 2015 Nov 2;15:832. doi: 10.1186/s12885-015-1869-6 (PMC4629406; doi:10.1186/s12885-015-1869-6)
Supplement: Additional file 5: Table S1. — Patients and Tumor Characteristics. (DOC 47 kb) [file 12885_2015_1869_MOESM5_ESM.doc]

**Supplementary Table S1 Patients** and Tumor Characteristics

|  | **β2-AR scores** | | | |  |
| --- | --- | --- | --- | --- | --- |
|  | **Low** | | **High** | | ***P* value** |
|  | **No. of Patients** | **%** | **No. of Patients** | **%** |  |
| **No. of patients** | 26 |  | 33 |  |  |
| **Age, years** |  |  |  |  |  |
| ≥45 | 16 | 61.54 | 22 | 66.67 |  |
| <45 | 10 | 38.46 | 11 | 33.33 | > 0.05 |
| **Post menopause** |  |  |  |  |  |
| Yes | 7 | 26.92 | 8 | 24.24 |  |
| No | 19 | 73.08 | 25 | 75.76 | > 0.05 |
| **Tumor size** |  |  |  |  |  |
| 1 | 1 | 3.85 | 3 | 9.09 |  |
| 2 | 22 | 84.62 | 20 | 60.61 | > 0.05 |
| 3 | 3 | 11.54 | 7 | 21.21 |  |
| 4 | 0 | 0 | 3 | 9.09 |  |
| **ER** |  |  |  |  |  |
| Positive | 11 | 42.31 | 14 | 42.42 |  |
| Negative | 15 | 57.69 | 19 | 57.58 | > 0.05 |
| **PR** |  |  |  |  |  |
| Positive | 9 | 34.62 | 11 | 33.33 |  |
| Negative | 17 | 65.38 | 22 | 66.67 | > 0.05 |
